# Physical activity and improvement of physiological parameters by training

**DOI:** 10.3389/fspor.2026.1944574

**Published:** 2026-09-28

**Authors:** Ricardo Augusto de Melo Reis, Ricardo Mario Arida, Antonio Claudio Lucas da Nobrega, João Dario Martins de Mattos, Hércules Rezende Freitas, Maria Clara Salgado Ramos, Rodrigo Soares Fortunato, Leonardo Matta, Gustavo Costa Ferreira, Cláudio Canetti

**Affiliations:** 1 Laboratory of Neurochemistry, Institute of Biophysics Carlos Chagas Filho, Universidade Federal do Rio de Janeiro, Rio de Janeiro, Brazil; 2 Departament of Physiology, Federal University of São Paulo, São Paulo, Brazil; 3 Laboratory of Exercise Sciences, Department of Physiology and Pharmacology, Fluminense Federal University, Niteroi, Brazil; 4 National Institute of Science and Technology Physical (In)Activity and Health, Niteroi, Brazil; 5 Departamento de Ciências Médicas Integradas, Faculdade de Ciências Médicas, Universidade do Estado do Rio de Janeiro, Rio de Janeiro, Brazil; 6 Laboratório de Fisiologia Endócrina Doris Rosenthal, Universidade Federal Do Rio de Janeiro, Rio de Janeiro, Brazil; 7 Laboratory of Inborn Errors of Metabolism, Institute of Medical Biochemistry Leopoldo de Meis, Universidade Federal do Rio de Janeiro, Rio de Janeiro, Brazil; 8 Laboratory of Inflammation, Biophysics Institute Carlos Chagas Filho, Federal University of Rio de Janeiro, Rio de Janeiro, Brazil

**Keywords:** diet, mitochondria, myokines, physical activity, quality of life, resistance training, VO_2_max

## Abstract

Living a better life greatly depends on maintaining healthy physiological parameters, including cardiovascular fitness, a well-balanced neuro-immuno-endocrine axis, and cognitive function—all of which contribute to focus, sharp memory, and self-esteem while mitigating age-related cognitive decline. Physical activity (high, moderate and resistance training, RT) is one of the most important lifestyle choices for reducing disease risk and improving overall health, particularly aerobic training, which increases maximal oxygen uptake (VO_2_max), a marker of cardiorespiratory fitness. Here, we focus on how exercise influences physiology that may contribute to improvements in health-related quality of life (QoL). Specifically, we discuss based on evidence from randomized controlled trials, observational studies, and experimental animal models the neurobiological, endocrine, immune, metabolic, and cardiovascular adaptations induced by exercise, including changes in neurochemical signaling, trophic mediators, hormones, cytokines, and cardiorespiratory fitness. Specific nutrients as supplements (creatine, omega-3 fatty acids and some amino acids) and dietary patterns (mediterranean, ketogenic, intermittent fasting) are also discussed. It is never too late to start moving—regular physical activity has been shown to counteract several deleterious effects of a sedentary lifestyle by reducing systemic inflammation, attenuating age-related brain changes, and lowering the risk of neurodegenerative diseases.

## Introduction

Evidence supports regular physical activity as a key non-pharmacological strategy for enhancing health and quality of life (QoL) throughout the lifespan (1). Recent research suggests that genetics may have a stronger influence on longevity than previously thought. Based on analysis of three Scandinavian twin cohorts, longevity appears to be approximately 50% heritable, particularly as individuals reach very old ages (2). However, diet and lifestyle factors do not affect people merely on a temporary basis, they can modify gene expression without altering the DNA sequence itself, inducing long-term, cumulative effects on health. These changes occur through epigenetic mechanisms such as DNA methylation and histone modifications that alter chromatin structure (3), essentially determining which genes are “turned on” or “off.” The concept of “heritable metabolic outcomes” is particularly intriguing: some epigenetic changes can potentially be passed to offspring, though this remains an active area of research in humans. This suggests that our lifestyle choices might influence not only our own health but potentially that of future generations.

QoL is defined as the subjective perception of life position in the context of culture and value systems in relation to individuals goals, expectations, standards and concerns (4). Alternatively, health-related quality of life (HRQoL) is a tighter definition including the areas of life connected by health status, disease, injury, or treatment (5). HRQoL was updated from the earlier Wilson and Cleary model as broad QoL parameters are often incompletely responsive to identify changes given to a specific health condition or clinical/behavioral intervention. The two most used instruments are WHOQOL (WHOQOL-100) that assesses global QoL across six domains—physical health, psychological, level of independence, social relationships, environment, spirituality/personal beliefs and SF-36 (36-Item Short Form Health Survey), the most widely applied generic HRQoL instrument in the exercise physiology literature. It includes eight specific health domains (6)—physical functioning, role limitations due to physical problems, bodily pain, general health, vitality, social functioning, role limitations due to emotional problems, and mental health—which sum into two scores, the Physical Component Summary (PCS) and Mental Component Summary (MCS).

Within this framework, physical activity has emerged as one of the most effective non-pharmacological strategies for improving HRQoL across different populations by promoting gains in physical function, psychological well-being, functional Independence, and social participation (7). Therefore, improvements in physiological parameters, such as aerobic capacity, muscle strength, glycemic control, or inflammatory biomarkers, should be interpreted as potential mechanisms or intermediate outcomes rather than direct evidence of enhanced HRQoL. Accordingly, throughout this review, physiological adaptations are interpreted as mechanistic pathways that may contribute to improvements in HRQoL, whereas conclusions regarding QoL are discussed only when supported by validated patient-reported outcome measures.

Key factors contributing to long-term health include diet, physical activity, sleep, smoking status, and alcohol consumption. Regular exercise, particularly aerobic training, increases maximal oxygen uptake (VO_2_max), a marker of cardiorespiratory fitness and higher levels are consistently associated with lower cardiovascular and all-cause mortality, particularly among metabolically compromised individuals (8).

High-Intensity Interval Training (HIIT) consists of repeated bouts of high-intensity exercise interspersed with recovery periods. In individuals with lifestyle-related cardiometabolic disorders, HIIT has been shown to produce substantial improvements in cardiorespiratory fitness and may yield greater increases in VO_2_max (9). Designed for efficiency, 10-to-30-minute HIIT workouts can substantially increase energy expenditure, improve cardiovascular health, increase metabolism, and drive physiological adaptations to endurance exercise training in both sedentary and recreationally trained individuals (10). Alternatively, Moderate-Intensity Continuous Training (MICT) is a cardiovascular workout modality involving continuous, uninterrupted activity (e.g., jogging, cycling, swimming) at a moderate pace, typically performed for 20–60 min. MICT requires maintaining 55%–75% of maximum heart rate or 40%–69% of peak capacity (9). Both HIIT and MICT are effective at improving metabolic markers, reducing glycated hemoglobin (HbA1c) and glycemic levels, lowering cardiovascular risk, and enhancing physical and psychological well-being. HbA1c is a crucial blood marker that quantifies average blood glucose over the preceding 2–3 months and serves as an indicator of prediabetes, type 2 diabetes, and long-term glucose control in patients (11). Resistance training (RT) is defined as activity that demands skeletal muscle to contract against an external load or resistance (free weights, machines, elastic bands, or body mass) with the goal of increasing muscular strength, power, endurance, or hypertrophy. Physiologically, it is defined by three core principles: progressive overload—the load must exceed habitual demand and increase over time to drive continued adaptation, specificity—adaptations (strength, power, hypertrophy, endurance) match the training variables used (intensity, volume, velocity) and mechanical tension + metabolic stress + muscle damage—the three proposed mechanistic drivers of hypertrophic and neuromuscular adaptation (12).

Combined aerobic and RT appears most effective overall, with HIIT associated with the greatest reduction in HbA1c (Figure 1).

**Figure 1:**
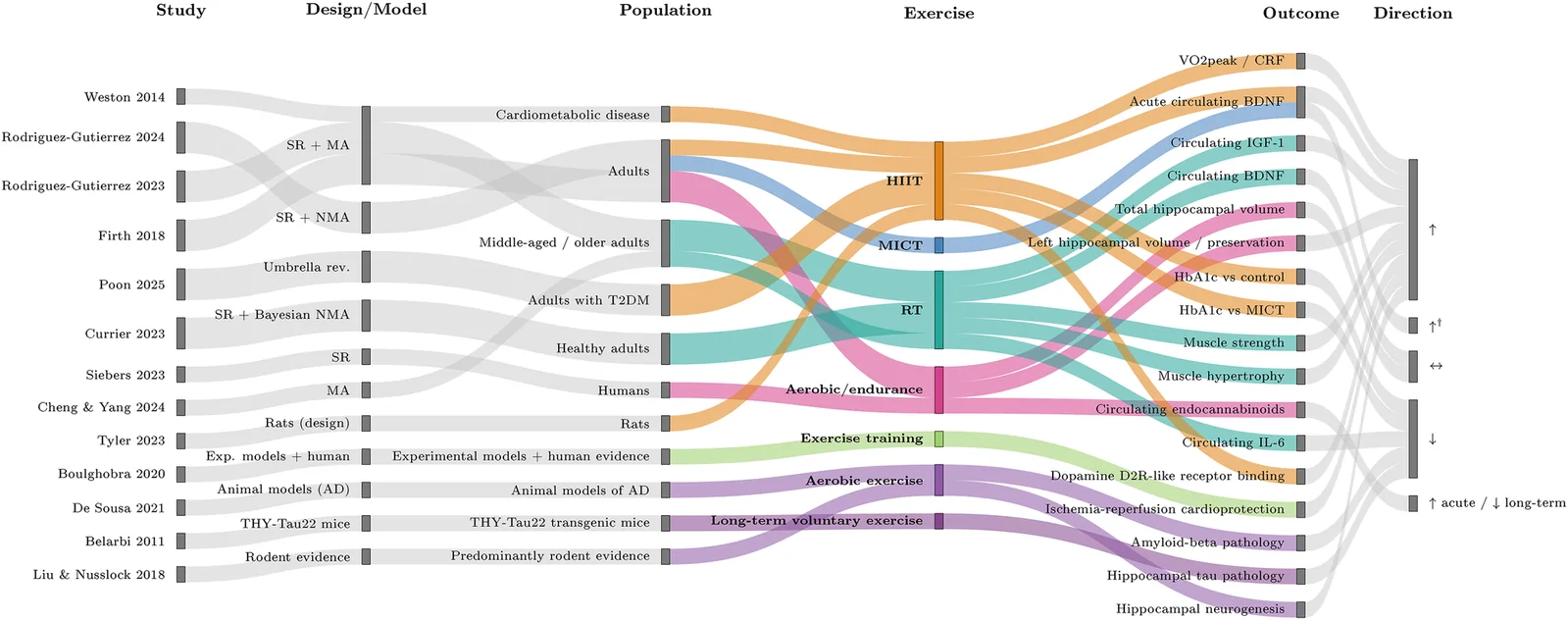
Exercise modality shapes distinct molecular, structural, and functional adaptations across human and preclinical evidence. Alluvial diagram linking each of the 13 sources included in this synthesis (contributing 18 study-to-outcome associations) to the exercise modality it examined and the outcome it reported. Flows trace, left to right, the study, its design or model, the population studied, the exercise modality applied, the resulting outcome measure, and the direction of change reported for that outcome. Ribbons are colored by exercise modality: HIIT, MICT, RT, aerobic/endurance training, and three lower-frequency categories from preclinical work (exercise training, aerobic exercise, long-term voluntary exercise). Grey ribbons carry no modality-specific meaning and simply indicate structural links between study, design, and population. Direction arrows follow each source's own classification: ↑ indicates an increase, ↓ a decrease, ↔ no significant change, and ↑ acute/↓ long-term a biphasic response reported over different time windows (13), circulating endocannabinoids). The dagger symbol (↑†) reproduces a qualifier from the original source (14), MICT; confirm its exact meaning against that source before using it in the final caption. Design/Model abbreviations are decoded beneath the figure. Design/Model abbreviations: SR + MA, systematic review + meta-analysis; SR + NMA, systematic review + network meta-analysis; Umbrella rev., umbrella review; SR + Bayesian NMA, systematic review + Bayesian network meta-analysis; SR, systematic review; MA, meta-analysis; Rats (design), rat model; Exp. models + human, experimental models + human evidence; Animal models (AD), animal models of Alzheimer's disease; THY-Tau22 mice, THY-Tau22 transgenic mice; Rodent evidence, predominantly rodent evidence.

In this narrative review, we aim to explore physical activity as one of the most important lifestyle choices for reducing disease risk and improving overall health. We followed SANRA (scale for the quality assessment of narrative review articles) (15) and we focus on how physical activity profoundly impacts QoL by improving physiology, neurochemical systems, trophic factors, myokines and hormones, improving cardiovascular parameters (VO_2_max), and reducing inflammation.

### Classical neurochemical systems and exercise

Physical activity induces neurochemical adaptations that may contribute to improvements in HRQoL by modulating mood, cognition, motivation, and emotional well-being (16). However, these neurochemical changes should be interpreted as biological mechanisms rather than direct measures of HRQoL. Several neurotransmitter systems are heavily involved in exercise, both driving performance and responding to it. The monoamine neurotransmitters dopamine (DA), norepinephrine (NE), and serotonin (5-HT) play crucial roles in brain function during exercise. DA is well known for its involvement in motivation, motor control, decision-making, and reward (17). Evidence from both human and experimental animal studies suggests that dopamine concentrations increase during and immediately after exercise, reinforcing motivation and reward-related behaviours. It also plays a central role in motor control and habit formation via the mesocorticolimbic system (18). Aerobic exercise, such as running, jogging, cycling, or swimming, raises DA levels in brain regions associated with habit formation, memory, and mood (19). Data from rodents clearly show mechanistic effects. For instance, chronic training upregulates DA receptor sensitivity. HIIT, compared to aerobic and resistance exercise in rats, showed a 16% increase in D2R-like dopaminergic receptor binding compared to sedentary rats (20). No significant effects were found on D1R-like receptor binding levels or tyrosine hydroxylase enzyme expression. These DA signaling changes could partially explain improved mood and motivation in regular exercisers.

Human observational studies and randomized exercise interventions suggest that increased serotonergic activity contributes to improved mood, reduced anxiety, better sleep quality, lower fatigue perception, and enhanced emotional well-being (21). As muscles consume branched-chain amino acids during exercise, tryptophan faces less competition crossing the blood-brain barrier (BBB), leading to greater 5-HT synthesis (22). This effect is more pronounced with moderate, sustained activity (such as a 20–40-min jog) rather than short bursts. Since 5-HT regulates mood, cognition, and stress responses, enhanced serotonergic neurotransmission represents one of the main biological mechanisms through which aerobic exercise may influence psychological well-being and HRQoL (21).

While primarily involved in the cardiovascular response to exercise, NE also plays a role in mood and alertness (23). NE is involved in metabolic responses to exercise, increasing oxygen and fuel supply to working muscles, causing vasoconstriction in non-working tissues, and promoting fat mobilization. It is essential for the “ready to perform” state (23). NE and DA also signal central fatigue during prolonged exercise, which combines with a faster rate of increase in the rating of perceived exertion (24). Mental fatigue affects several neurotransmitter systems, with presumably important roles for adenosine in multiple brain regions such as the prefrontal cortex (PFC) and anterior cingulate cortex.

Other classical neurotransmitters, glutamate, GABA, and acetylcholine may also be involved with brain motor systems during exercise. Glutamate is the principal excitatory neurotransmitter in the motor cortex and spinal cord, coordinating voluntary movement. Glutamate and GABA, the main inhibitory neurotransmitter in the central nervous system, may also be acutely modulated by exercise. In healthy human volunteers, proton magnetic resonance spectroscopy demonstrated significant increases in both glutamate and GABA signals in the visual cortex following vigorous exercise, together with an increase in glutamate in the anterior cingulate cortex (25). These findings provide direct human evidence that acute physical exercise can alter cortical glutamate and GABA pools; however, changes in MRS-measured metabolite concentrations should not be interpreted as equivalent to changes in synaptic neurotransmission or GABAergic tone.

Most of the cholinergic cells in the spinal cord are motoneurons that innervate skeletal muscles, with their cell bodies distributed within the ventral gray matter, reviewed in Le Ray et al. (26). Acetylcholine is found primarily at the neuromuscular junction (NMJ), triggering every muscle contraction by motor axons during exercise. Exercise may influence cholinergic function through both central and peripheral mechanisms. At the peripheral level, the NMJ represents an important site of exercise-induced adaptation. Experimental and human studies indicate that exercise can induce structural and functional adaptations at the NMJ. In humans, RT has been associated with changes in NMJ morphology (27), whereas evidence regarding nerve regeneration and recovery following peripheral nerve injury derives predominantly from experimental models (28).

### Trophic factors, neuroplasticity and exercise

Evidence from randomized controlled trials, observational studies, and experimental animal models consistently demonstrates that chronic exercise (including aerobic and endurance training) increases the release of several neurotrophic, most notably brain-derived neurotrophic factor (BDNF), one the most extensively studied in both the brain and peripheral circulation (29). Experimental studies demonstrate that BDNF promotes neuronal survival, synaptic plasticity, and hippocampal neurogenesis, whereas human studies have associated higher circulating BDNF concentrations with improved cognitive performance and reduced depressive symptoms (30). Experimental evidence suggests that locally synthesized IGF-1 contributes to protection against cerebral ischemia. IGF-1 also promotes skeletal muscle repair and may contribute to regulation of neuroinflammatory responses (31). Both experimental and human studies indicate that exercise increases vascular endothelial growth factor (VEGF) expression, which promotes angiogenesis, the formation of new blood vessels in the brain. This improves cerebral blood flow and oxygen delivery, supporting cognitive function and neuroplasticity.

Two additional trophic factors, Fibroblast Growth Factor 2 (FGF-2) and GDNF, have been shown to increase following chronic exercise and are involved in stem cell proliferation, neurogenesis, and neuroprotection. FGF-2 may play a role in stress resilience and maintenance of dopaminergic neurons, making it relevant to Parkinson's disease. Exercise-induced GDNF may partially explain the protective effects of physical activity against neurodegenerative conditions (32). These factors work synergistically and appear to be interconnected and triggered by exercise. Indeed, several myokines (IGF-1, Irisin) have been shown to protect against neuronal injury in cerebral ischemia models, suggesting that trophic factors may influence neuronal damage in part by inhibiting inflammatory signaling pathways (Figure 2).

**Figure 2:**
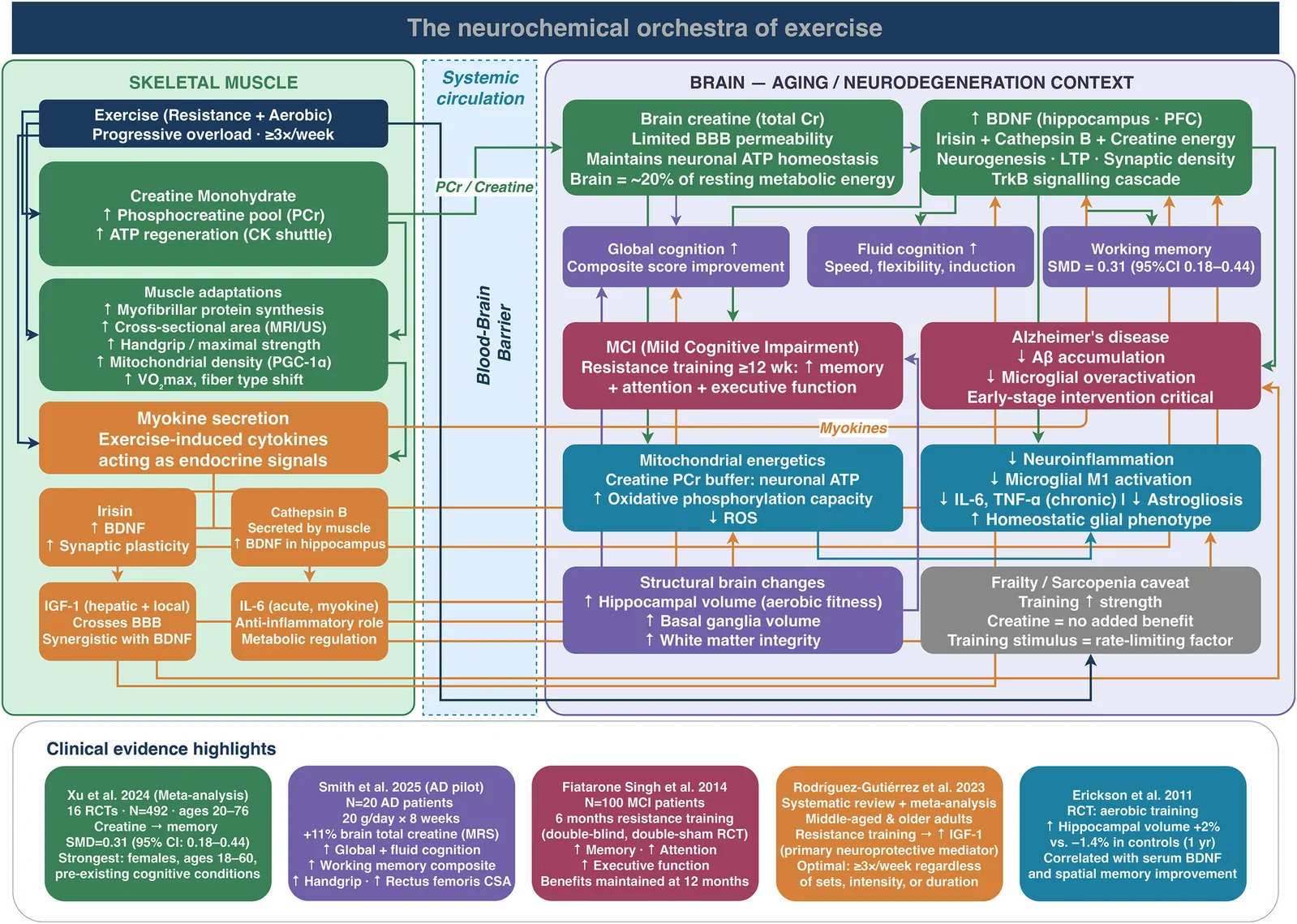
Exercise involving combined resistance and aerobic modalities (≥3×/week) activates a coordinated cascade of peripheral and central neurochemical events. In skeletal muscle (**left panel**), resistance training in combination with creatine monohydrate supplementation expands the phosphocreatine pool (PCr) and increases ATP regeneration via the creatine kinase shuttle. This allows myofibrillar protein synthesis, mitochondrial biogenesis (PGC-1α), and VO_2_ max improvement. Exercise-induced myokine secretion—mainly irisin, cathepsin B, IGF-1, and acute IL-6—constitutes an endocrine axis that connects both the PNS and CNS via the systemic circulation. Irisin and cathepsin B are proposed to cross the BBB and upregulate BDNF in the hippocampus and PFC, activating TrkB signaling and downstream cascades that promote neurogenesis, long-term potentiation (LTP), and synaptic density. In the brain (**right panel**), the neurochemical effects of exercise are particularly relevant in the context of aging and neurodegeneration: aerobic training increases hippocampal and basal ganglia volume and white matter integrity, while RT is proposed to drive IGF-1-mediated neuroprotection and attenuate Aβ accumulation and microglial overactivation, and preserves the homeostatic glial phenotype. Creatine contributes to neuronal ATP homeostasis by supporting brain phosphocreatine buffering capacity, leveraging the fact that the brain consumes approximately 20% of resting metabolic energy. Clinical evidence panels (**bottom**) summarize key human trials supporting each pathway. Boxes and connectors summarize relationships of heterogeneous evidential status and are not intended as a unified causal model. The peripheral adaptations (phosphocreatine and creatine kinase flux, myofibrillar protein synthesis, mitochondrial density, VO_2_ max) and the cognitive outcomes shown in the brain panel derive from randomized controlled trials and meta-analyses in humans, five of which are summarized in the clinical evidence panels at the bottom. The myokine limb, comprising irisin, cathepsin B and cathepsin B-dependent hippocampal BDNF induction, rests primarily on rodent and *in vitro* work: transfer of these myokines across the blood-brain barrier and their causal contribution to human cognitive gains are proposed rather than demonstrated. Connectors indicate the direction of a hypothesized relationship, not its strength or its degree of experimental confirmation.

### Endocannabinoids and endorphins

Evidence from randomized controlled trials and meta-analyses indicates that regular exercise exerts clinically meaningful antidepressant and mood-enhancing effects while contributing to stress reduction. The phenomenon known as the “runner’s high” was initially attributed primarily to endogenous opioid release, particularly β-endorphin; however, more recent experimental evidence indicates that endocannabinoids play a central role in mediating this response (13, 33). Unlike endorphins, which are large molecules that struggle to cross the BBB, endocannabinoids enter the brain where they bind to cannabinoid CB_1_/CB_2_ receptors. The two most well-known endocannabinoids are anandamide (AEA) and 2-arachidonoylglycerol (2-AG). Experimental and human studies suggest that AEA readily crosses the BBB and is a major contributor to exercise-induced euphoria, analgesia, anxiolysis and exercise-related well-being during running and cycling (13). 2-AG also penetrates the BBB with an important role in the protection and maintenance of its integrity, limiting inflammation and damage mediated through CB₁/CB_2_ receptors (34). Endocannabinoids respond differently to various exercise intensities. During HIIT, the body releases AEA and 2-AG, produced by peripheral tissues (muscle, fat) and the brain, which act on CB₁ receptors (predominant in the CNS) and CB_2_ receptors (predominant in the periphery). High-intensity exercise is associated with acute increases in circulating AEA concentrations; however, the magnitude of this response depends on exercise intensity, duration, fitness level, and exercise modality rather than a fixed heart-rate threshold (35). A randomized crossover study in trained male cyclists demonstrated that acute exercise, especially when intense, induces a large increase in circulating levels of AEA, which persists during recovery, possibly through mechanisms involving cortisol. This suggests that a synergistic effect with BDNF, key to hippocampal neurogenesis, long-term potentiation (LTP), and memory formation may be a mechanism by which AEA/CB₁ signaling influences the neuroplastic and antidepressant effects of exercise (35). These findings may contribute to a positive feedback mechanism whereby rewarding exercise experiences facilitate long-term adherence to physical activity. Other mechanisms are involved, including an increase in cerebral blood flow, with more oxygen and glucose to the hippocampus and prefrontal cortex (PFC); in addition, IGF-1 and VEGF (critical for cerebral angiogenesis) reduce neuroinflammation (protection against cognitive decline). Large observational studies and international guidelines consistently indicate that regular moderate-to-vigorous physical activity is associated with improvements in multiple metabolic, cardiovascular and immune outcomes (36). Scientific evidence supports that MICT (running/cycling) is highly effective at elevating circulating endocannabinoid levels, specifically AEA and 2-AG (37). Individuals who exercised on a treadmill or stationary bike for 6 months had increased levels of AEA in the bloodstream when compared to sedentary individuals. This typically occurs during sustained activities. Experimental and clinical evidence supports a bidirectional interaction between endocannabinoids and neurotrophic factors that contributes to brain health and plasticity (35, 38) with positive effects on cognitive functions and hippocampal plasticity (Figure 3) through a dual pathway: (a) a direct pathway, where intense exercise is the most efficient stimulus for releasing BDNF, which promotes neurogenesis in the dentate gyrus and an indirect pathway, (b) involving endocannabinoid, which act on CB1 receptors increasing BDNF release—AEA activates CB1 in the hippocampus, which in turn upregulates BDNF expression, a synergistic loop.

**Figure 3:**
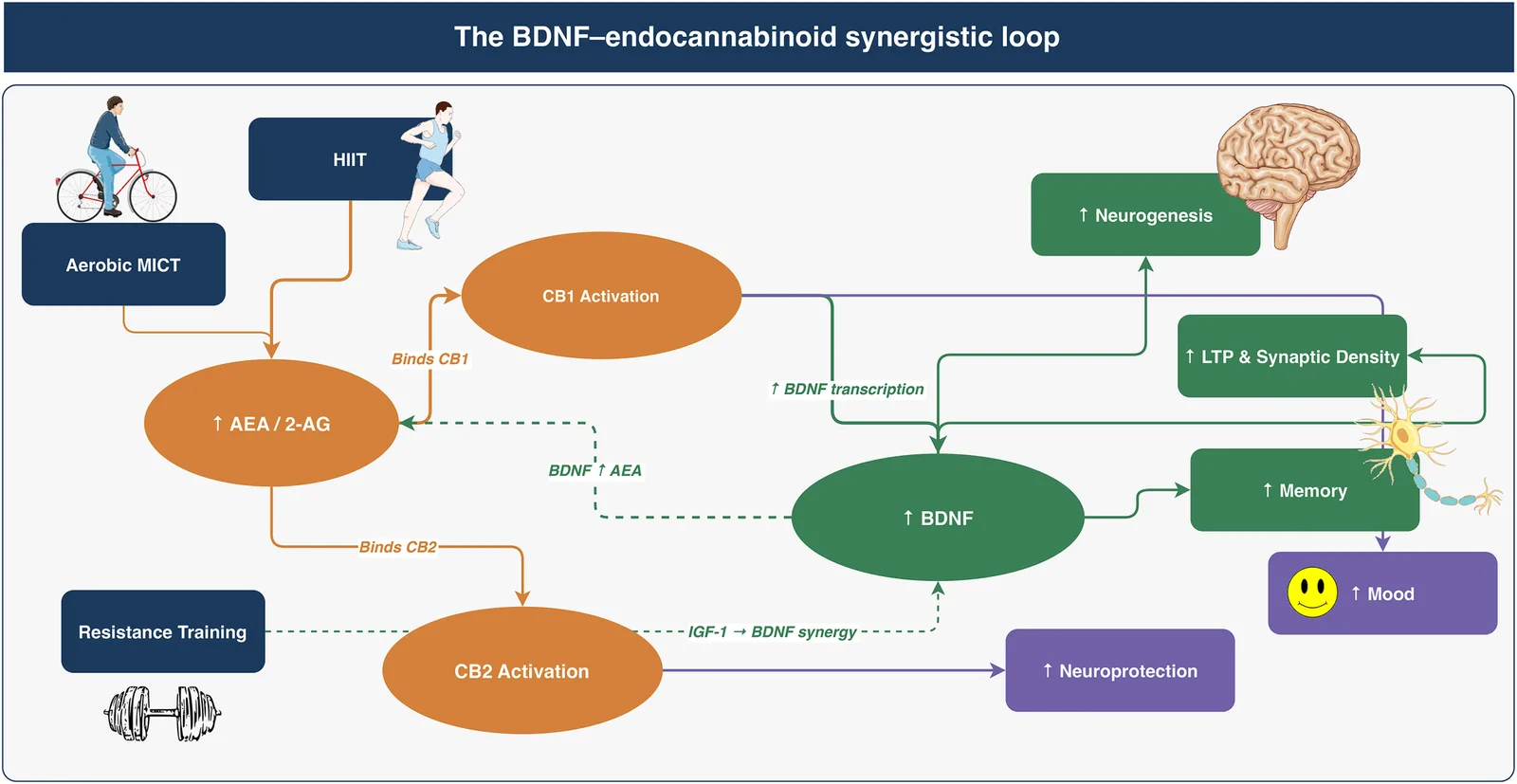
The BDNF–endocannabinoid synergistic loop: exercise-induced neuromodulation through convergent molecular pathways. This figure illustrates the bidirectional, self-reinforcing interaction between brain-derived neurotrophic factor (BDNF) and the endocannabinoid system (ECS) following different exercise modalities. High-intensity interval training (HIIT) at intensities of 70%–85% of maximal heart rate, or higher, acutely elevates circulating anandamide (AEA) and 2-arachidonoylglycerol (2-AG), released from muscle, adipose, and brain tissues. AEA binds cannabinoid receptor type 1 (CB1) in the hippocampus and prefrontal cortex, which in turn upregulates BDNF transcription. In a positive feedback loop, BDNF reciprocally enhances ECS tone by increasing AEA precursor availability. Aerobic moderate-intensity continuous training (MICT; 20–60 min sustained) preferentially elevates tryptophan and serotonin (5-HT), while RT augments IGF-1 and irisin through the muscle-brain axis. The convergent downstream outcomes include enhanced hippocampal neurogenesis, increased LTP and synaptic density, hippocampal volume expansion, and improved spatial, working, and object memory consolidation. Neuroprotective effects include reduced Aβ plaque burden and tau pathology through microglial regulation. Rodent pharmacological studies, not depicted in the diagram, show that CB1 blockade (AM251) abolishes exercise-induced BDNF upregulation and memory enhancement, while ECS tone mimicry (URB597) replicates training benefits in sedentary animals. The cascade linking AEA-CB1 signaling to BDNF transcription derives predominantly from rodent and *in vitro* work and is presented as a proposed mechanism. Human studies support exercise-induced elevations in circulating endocannabinoids and in peripheral BDNF, but the bidirectional loop as a whole has not been demonstrated in humans, and the two dashed connectors in the diagram (BDNF to AEA availability, and IGF-1 to BDNF synergy) mark the limbs that remain hypothetical. The pharmacological findings obtained with AM251 and URB597 come from rodent experiments and are reported here for context; they are not depicted in the diagram.

Beyond its neurobiological effects, exercise also influences psychosocial dimensions closely related to HRQoL. Furthermore, regular exercise may improve self- esteem through favorable changes in body composition, physical function, and self-perception. Achieving exercise-related goals, even modest ones, may also enhance self- efficacy, the belief in one’s ability to successfully perform specific tasks, which is an important determinant of long-term exercise adherence and overall well-being (39).

### How exercise influence brain health?

Several factors contribute to the beneficial effects of exercise on the brain and different forms of physical exercise have been examined through physiological, psychological, and biochemical approaches within the field of neuroscience. Investigating the physiological effects of exercise on the human brain presents significant challenges due to the high invasiveness of the data-collection techniques required. Consequently, direct observation of exercise-induced changes in brain physiology is most often conducted using animal models. In this context, the impact of different types of physical exercise across the lifespan, from brain development to aging will be considered.

### Physical exercise in pregnancy and its influence on the offspring brain

Human brain development begins in the third gestational week and continues at least through late adolescence. This process extends from the molecular changes of gene expression to the complex effects of environmental stimulation. In this line, studies have shown that environmental stimuli, particularly physical activity and exercise, can positively impact brain development. In humans, only a limited number of studies have examined whether exercise during the maternal period can influence the offspring's brain. In a classic study conducted over 30 years ago, a better performance on tests of general intelligence and oral language skills was observed in 5-year-old children whose mothers exercised during pregnancy (40). In another study, maternal physical activity before and during gestation was associated with better academic performance in math and language in offspring aged 6–18 years (41).

Although the contribution of pre-natal exercise to brain health in offspring has poorly been explored in humans, promising data have been provided in animal studies. Findings from animals have demonstrated that exercise during gestational period can improve the pup's brain functions using different models of physical exercise, including voluntary wheel running, forced treadmill running and forced swimming. For example, swimming or treadmill running during pregnancy enhances offspring spatial learning (42) and short-term memory (43) and voluntary running mitigates depression-like behaviors and improves cognitive performance (44). Several factors may contribute to these effects. Both forced and voluntary maternal exercise positively influence cell proliferation and survival during rodent development. Also, both exercise types from prenatal day 15–21 increased the number of BrdU labeled cells on post-natal day 29 in the hippocampus, a region related to learning and memory, which was associated with an improvement of cognitive function (43, 45). Interesting, exercise during pregnancy also induces changes in later life. Treadmill running led to an increased number of neuronal and non-neuronal cells and BDNF levels in the hippocampal formation of offspring at 60 days after birth (46) and cell density in the hippocampal CA1 and CA3 regions of adult offspring at postnatal day 120 of offspring of mothers exercised during pregnancy (47).

Regarding strength exercise, there are very few studies in the literature demonstrating the effects of resistance exercise during gestation on the offspring's brain. Resistance exercise during gestation was associated with enhanced cell proliferation in the dentate gyrus and reduced global DNA methylation in the offspring's hippocampus during the postnatal period (P12–P21) (48). A subsequent study by the same research group showed that offspring of mothers who exercised exclusively during pregnancy exhibited subtly improved performance on memory and learning tasks, along with decreased hippocampal Histone deacetylase 2 (HDAC2) levels. Thus, offspring of mothers who exercised before the gestational period showed increased cell proliferation and elevated IGF-1 expression in the hippocampal dentate gyrus, as well as alterations in global DNA methylation and H4 acetylation (49). Therefore, it is suggested that physical exercise during the gestational period may produce enduring effects and, consequently, contribute to increased positive brain plasticity in the offspring. Although exercise is generally recommended during pregnancy, certain medical or obstetric conditions may require modifications or restrictions. Consequently, all exercise programs should be planned and monitored in consultation with a healthcare provider, in accordance with the American College of Obstetricians and Gynecologists Guidelines for exercise during pregnancy and the postpartum period (50).

### Physical exercise during childhood and adolescence

Investigations in both humans and animal models have shown that physical exercise can exert significant effects on the brain during postnatal development. In humans, exercise during this period has been associated with improvements in reading comprehension, language processing, attention, and performance in arithmetic tasks (51). Some of the neurobiological bases underlying these benefits include increase of cortical activity, white matter integrity, and gray matter microstructure (52). Other contributing factors include the release of BDNF, IGF-I, and VEGF (Figure 4). Beyond their direct neurotrophic actions, these molecules also support synaptogenesis, angiogenesis, metabolic regulation, and activity-dependent plasticity, thereby contributing to the structural and functional maturation of neural circuits. A systematic review and meta-analysis highlight the effects of physical exercise on neurotrophins levels and executive functioning in adolescents (53).

**Figure 4:**
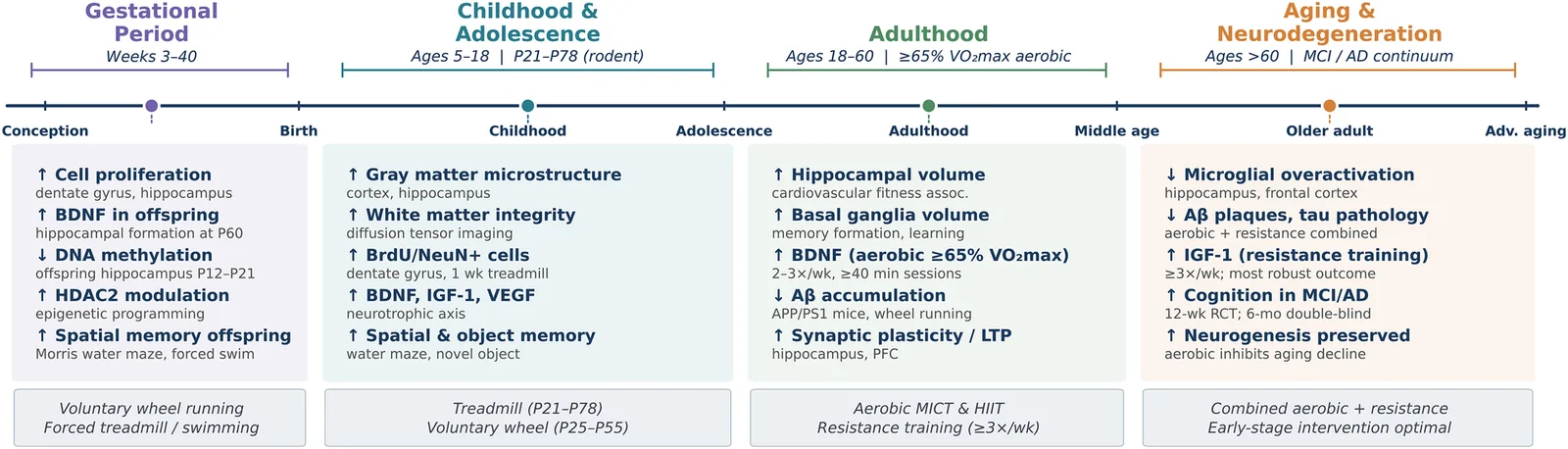
Physical exercise and brain health across the lifespan: sensitive windows, neurobiological mechanisms, and evidence-based exercise models. A chronological timeline spanning conception through advanced aging depicts the major sensitive windows during which physical exercise exerts documented neurobiological effects. During the gestational period (weeks 3–40), voluntary wheel running and forced treadmill exercise in pregnant dams increases cell proliferation in the dentate gyrus and hippocampus, elevates offspring BDNF levels at postnatal day 60 (P60), reduces DNA methylation in the offspring hippocampus (P12–P21), and enhances spatial memory performance in offspring. During childhood and adolescence (ages 5–18; P21–P78 in rodents), exercise promotes gray matter microstructure in the cortex and hippocampus, improves white matter integrity as measured by diffusion tensor imaging, increases BrdU/NeuN + cell counts in the dentate gyrus following treadmill training, and upregulates the neurotrophic axis (BDNF, IGF-1, VEGF). In adulthood (ages 18–60), aerobic exercise at ≥65% VO_2_ max for ≥40 min performed 2–3× sessions/week is the most effective stimulus for hippocampal and basal ganglia volume expansion, BDNF elevation, and synaptic plasticity enhancement; RT additionally attenuates Aβ accumulation in APP/PS1 mouse models. In aging and neurodegeneration (ages >60; MCI/AD continuum), combined aerobic and RT reduces microglial overactivation, Aβ plaques, and tau pathology, with early-stage intervention yielding the most robust outcomes across 12-week RCTs and 6-month double-blind trials. The gray boxes below each window indicate the exercise models with the strongest supporting evidence for each life stage. Findings differ in provenance and are labelled accordingly within each window. Gray- and white-matter imaging, the adult and older-adult exercise prescriptions, and the 12-week and 6-month randomized trials come from human studies. BrdU/NeuN + cell counts, hippocampal DNA methylation and HDAC2 modulation, Morris's water maze and forced-swim performance, and APP/PS1 amyloid data come from rodent models. The gestational window is drawn on the human timeline (weeks 3–40) for comparability, but every finding listed within it was obtained in pregnant rodents and is mapped onto the human timeline by developmental equivalence rather than by direct measurement. Rodent findings indicate candidate mechanisms and are not evidence of equivalent effects in humans.

Similar outcomes are observed in animal studies using diverse exercise models. Treadmill running from postnatal day 22–78 (P22–P78) (54) or from P21 to P60 (55) produced superior spatial learning in the Morris water maze compared with controls. Likewise, voluntary wheel running from P25 to P55 improved the performance and increased the discrimination ratio between novel and familiar objects, a non-spatial measure of learning and memory, compared with non-exercising animals (56). For a comprehensive review, see Gomes da Silva and Arida (57). Increases in both neuronal and non-neuronal cell populations are proposed mechanisms underlying these benefits. For instance, a significant increase in BrdU/NeuN double-labeled cells in the dentate gyrus of the hippocampal formation was reported in adolescent rats subjected to one week of treadmill exercise (58, 59). Overall, regular physical exercise during this critical window of brain development may contribute to improvements in memory function and, consequently, to other aspects of academic performance. Although the underlying mechanisms have been characterized primarily in animal models, current evidence suggests that regular physical exercise during this critical developmental period contributes to cognitive maturation and academic performance.

### Physical exercise during adulthood and aging

As observed during early developmental stages, including childhood and adolescence, the adult human brain can also adapt structurally and/or functionally in response to environmental stimuli, such as physical exercise. Indeed, physical exercise during the adulthood is widely recognized as a protective factor against cognitive decline and stress-related outcomes, particularly among middle-aged and older adults. These benefits are mediated by the brain's capacity for reorganization and the formation of new neural connections, including increased production of neurotrophic factors, modulation of neuroinflammatory processes, enhancement of synaptic plasticity and neurogenesis (60). In the adult brain, various forms of physical exercise exert distinct effects on neuroplasticity through diverse molecular and cellular mechanisms, thereby offering a range of potential interventions to mitigate cognitive decline in neurodegenerative disorders.

Studies involving aerobic exercise show that higher levels of cardiovascular fitness are associated with differences in the volume of brain structures, particularly the hippocampus (61, 62) and the basal ganglia (63), both critically involved in memory formation and learning. These structural changes have been linked to increased secretion of peripheral neurotrophic substances. A systematic review and meta-analysis by Feter et al. (64), which investigated the dose–response relationship between BDNF concentration and physical exercise in humans, suggested that aerobic training performed at intensities ≥65% of VO_2_ max, two to three times per week, with sessions lasting at least 40 min, yields more favorable effects. With respect to cognitive performance, low-intensity aerobic exercise programs have demonstrated substantial impact for cognitive health in healthy older adults, such as significant improvements in spatial memory and mood regulation. Importantly, these benefits also extend to individuals with cognitive impairments (65). Overall, the benefits of aerobic exercise have been documented in several meta-analyses and reviews, including among individuals with Alzheimer's disease (AD), non-AD dementias and mild cognitive impairment (MCI) (66–68).

From a neuropathological standpoint, AD is characterized by several hallmark features, including the extracellular accumulation of β-amyloid (Aβ) in neuritic plaques and the formation of intracellular neurofibrillary tangles (NFTs) composed of hyperphosphorylated tau protein. In humans, the effects of aerobic exercise on Aβ and NFTs levels have been investigated using several measures in the brain, cerebrospinal fluid, and blood; however, the literature has shown inconsistent findings [for review see Rodriguez-Ayllon et al. (69)]. These limited and less consistent data likely reflect variations in study design, population characteristics, and Aβ measurement.

While aerobic exercise has been extensively studied, recent evidence demonstrates the impacts of resistance exercise on cognitive health. Indeed, the literature has demonstrated that increases in muscle activity induced by resistance exercise are often accompanied by neuroplastic adaptations in the brain. Resistance exercise has been increasingly recognized as a potential strategy for the prevention and treatment of MCI and AD, with recent studies demonstrating its efficacy in enhancing cognitive function and memory in these populations. For instance, a randomized controlled trial showed that a 12-week resistance exercise program improved cognitive function in older adults with MCI and highlighted training frequency, intensity, and duration as critical determinants of these benefits (70). Similarly, a randomized, double-blind, double-sham–controlled trial demonstrated that 6 months of resistance exercise in 100 patients with MCI induced significant improvements in memory, attention, and executive function. These positive effects were maintained for up to 12 months after the intervention (71).

Resistance exercise, like aerobic exercise, also stimulates the secretion of peripheral neurotrophic factors. Although increased BDNF levels have been reported in older adults (72), a recent systematic review and meta-analysis with middle-aged and older adults, which investigated the effect of resistance exercise on several neurotrophic factors such as IGF-1, BDNF, and VEGF, demonstrated that this neuroprotective effect was observed mainly through its influence on IGF-1. In their study, this outcome was more pronounced when training was performed at least three times per week, regardless of the number of sets, exercise intensity, session duration, or overall program length (73).

Experimental evidence from rodent models corroborates findings in humans, indicating that beneficial effects observed with both aerobic and resistance exercise are mediated by reduced amyloid deposition and phosphorylated tau accumulation, increased BDNF expression, and attenuation of neuroinflammation, for a review, see McGurran et al. (74). Aerobic exercise interventions, including treadmill training (75) and voluntary wheel running (76), significantly reduce brain Aβ accumulation in APP/PS1 transgenic mice. In the same way, resistance exercise reduces both the volume and number of Aβ plaques and attenuates tau pathology in the brain (77–79). However, another study observed a reduction in Aβ levels in resistance-trained, but not aerobically trained, AD mice (80). These findings suggest that combining resistance exercise with other forms of physical exercise may represent a more effective therapeutic strategy for reducing Aβ.

The beneficial effects of aerobic and resistance exercise may be in part, through the modulation of microglial activity (78, 79) and the reduction of astrogliosis (78). Microglia play essential role in the regulation of Aβ and hyperphosphorylated tau, and both astrocytes and microglia are markedly activated in brain regions associated with the initiation of AD pathology. Upon activation, these glial cells transition from a resting to a pro-inflammatory state and initially exert protective effects by clearing damaged proteins and cellular debris. However, prolonged exposure to injurious stimuli leads to sustained glial activation, resulting in excessive release of pro-inflammatory mediators that can disrupt cellular homeostasis and exacerbate neurodegeneration. In this regard, aerobic exercise has been shown to inhibit excessive activation of hippocampal microglia in aged mice, preserve nervous system homeostasis, and effectively prevent neuronal damage during aging (81, 82). In parallel, resistance exercise exerts comparable anti-inflammatory effects by reducing microglial activation in the frontal cortex and hippocampus (78). Conversely, other studies from AD animal models indicates that aerobic exercise does not consistently reduce Aβ plaque burden (83) with some studies reporting no effect (83) or benefits limited to younger animals (84). Likewise, resistance exercise appears to confer benefits predominantly at early disease stages, with improvements in memory deficits and Aβ levels restricted to the initial phase of AD (85).

While many studies report consistent cognitive benefits using aerobic and resistance exercise, not all have observed corresponding reductions in Aβ or phosphorylated tau levels. These discrepancies may reflect differences in the timing of intervention, as exercise initiated after extensive plaque formation appears less effective at reversing established pathology or fully restoring cognitive function. In contrast, exercise introduced during early disease stages or prior to plaque accumulation can slow pathological progression and more effectively preserve cognitive performance.

## Cardiovascular fitness and exercise

Just as neurochemical adaptations integrate as a network the central and periphery, the cardiovascular system participates in the same bidirectional loop, with cardiac and vascular adaptations both responding to and reciprocally reinforcing the neural and skeletal-muscle demands imposed by training.

It is well established that exercise reduces the risk of cardiovascular disease, metabolic disorders, and cancer, as well as premature mortality while simultaneously improving functional capacity and psychological well-being (86, 87). These benefits result from complex physiological mechanisms and clinical context involving cardiovascular, metabolic, and autonomic adaptations that occur in response to repeated exposure to physical exercise (88, 89). As defined by Centers for Disease Control and Prevention (CDC, 2011), exercise is a “physical activity performed during leisure time with the primary purpose of improving or maintaining physical fitness, physical performance, or health” and may be considered as a structured physiological stimulus that transiently challenges homeostasis, thereby promoting integrated adaptive responses across multiple physiological systems (90).

Acute responses reflect the rapid physiological adjustments necessary to sustain muscular work, whereas chronic adaptations encompass structural and functional remodeling that increases the organism's tolerance to repeated physiological stress (91, 92). Physical exercise involves dynamic or static muscular contractions that increase metabolic demand and energy expenditure. During exercise, skeletal muscle contraction rapidly elevates the requirement for oxygen and metabolic substrates, such as glucose and free fatty acids, requiring coordinated adjustments of the cardiovascular, respiratory, and metabolic systems, which sustain the energetic demand of muscular work (93). These responses primarily include increased cardiac output, redistribution of blood flow to active muscles, elevated pulmonary ventilation, and activation of metabolic pathways responsible for ATP resynthesis (90). Such adjustments represent acute physiological responses that allow the organism to maintain homeostasis in the face of increased energetic demand and heat production imposed by exercise (88).

From a cardiovascular perspective, cardiac output is augmented via increases in heart rate and stroke volume, whereas peripheral vasodilation promotes the redistribution of blood flow toward active skeletal muscle. These responses ensure adequate oxygen delivery and metabolic exchange during muscular work (88, 94, 95). Neural control mechanisms play a fundamental role in coordinating these cardiovascular responses. Parasympathetic withdrawal and sympathetic activation modulate heart rate, myocardial contractility, and vascular tone during exercise. The integration of neural signals originating from central command and peripheral afferent feedback ensures appropriate cardiovascular adjustments to exercise intensity (96, 97). Additionally, experimental investigations have demonstrated that baroreflex mechanisms remain functionally active during exercise but operate around a reset arterial pressure level, allowing appropriate regulation of blood pressure despite increases in cardiac output and metabolic demand (96, 98). When these stimuli are applied repeatedly and systematically, as in physical training, they trigger adaptive processes that enhance the organism's functional efficiency. In this context, training organization is based on fundamental principles such as overload, specificity, individual variability, progression, and reversibility, which guide appropriate exercise prescription to promote positive physiological adaptations (65, 99). The specificity principle indicates that adaptations are specific to the type and muscle groups involved, while the overload principle states that the organism must be exposed to stimuli beyond habitual levels to induce adaptation, considering the training load as the product of intensity, duration, and frequency (sessions per week/month) of the applied stimulus. The gradual progression of training load and respect for individual characteristics ensure safe and efficient adaptation to exercise-induced stress. Thus, physical training represents a planned process of exposure to physiological stress that promotes metabolic, cardiorespiratory, and neuromuscular adaptations, contributing to improvements in functional capacity, cardiorespiratory fitness, and overall health (100, 101). Whereas adaptation to exercise training is known to occur in all individuals, including the elderly and patients with all kinds of chronic diseases, exercise protocol should be ideally tailored according to age, clinical status, and personal goals.

One of the most recognized cardiovascular adaptations to endurance training is resting bradycardia, which reflects increased vagal modulation and greater stroke volume in trained individuals (102, 103). In addition, trained individuals typically exhibit lower heart rate responses during submaximal exercise, indicating improved cardiovascular efficiency (104). Seminal studies conducted by Professor Peter Raven's group (105) demonstrated that exercise training can significantly modify autonomic modulation of heart rate. These findings indicate that cardiovascular adaptations to exercise training are strongly mediated by neural mechanisms, in which enhanced vagal modulation and attenuated sympathetic drive contribute to lower resting heart rate and improved cardiac efficiency.

At the peripheral level, exercise training induces important skeletal muscle adaptations. Increased capillary density enhances oxygen delivery to muscle fibers, while mitochondrial biogenesis improves oxidative metabolism and energy production (100, 106). These changes contribute to improvements in VO_2_max, considered the primary indicator of cardiorespiratory fitness. Mitochondria are the main energy-producing organelles, supplying over 80% of cellular ATP through aerobic respiration. During physical activity, oxygen consumption increases significantly due to the need of rapid ATP synthesis to sustain muscle contractions and maintain heart function (107). Mitochondria oxidize a variety of fuels to meet these heightened demands, including glucose, fats, and lactate. Mitochondrial fuel flexibility is a key component of the dynamic adaptions during exercise (108).

Indeed, cardiorespiratory fitness is a strong predictor of cardiovascular health and mortality. Epidemiological studies have demonstrated that individuals with higher levels of aerobic fitness exhibit significantly lower risks of cardiovascular disease and all-cause mortality (109–111). Increases in VO_2_max, result from both central and peripheral physiological adaptations. Central adaptations include increased stroke volume and cardiac output, while peripheral adaptations involve enhanced oxygen extraction by skeletal muscle (112, 113). In this context, skeletal muscle mass is closely associated with ventilatory and hemodynamic responses during exercise. Higher levels of cardiorespiratory fitness reduce the relative physiological effort required to perform daily activities, promoting greater functional independence and autonomy throughout life (114).

Exercise training also contributes to improvements in body composition and metabolic health. Although exercise alone does not always produce substantial weight loss without caloric restriction, it promotes reductions in adipose tissue and preservation or increases in lean muscle mass (115, 116). These changes are associated with improved metabolic regulation, including enhanced insulin sensitivity and reduced cardiometabolic risk (117, 118). Therefore, regular physical activity represents an important strategy for preventing metabolic diseases such as obesity and type 2 diabetes (1).

Regular physical activity plays a fundamental role in healthy aging. Aging is associated with declines in cardiovascular function, muscle mass, and physical capacity; however, exercise training can attenuate many of these changes (114). Exercise improves muscular strength, balance, and coordination in older adults, reducing the risk of falls and contributing to the maintenance of functional independence (119, 120). By increasing aerobic capacity and muscular strength, exercise allows daily activities to be performed with lower relative physiological stress, contributing to greater autonomy and improved HRQoL (7, 92, 119).

Finally, beyond physiological effects, regular physical activity also contributes to psychological and social well-being. Exercise participation has been consistently associated with improved self-esteem, better mood regulation, and reduced symptoms of anxiety and depression (121, 122). Additionally, physical activity may promote social interaction and community engagement, factors associated with social well-being and perceived well-being (123, 124). These multidimensional benefits reinforce the concept that the health effects of exercise extend beyond disease prevention, positively influencing physical, psychological, and social domains throughout the lifespan.

## Endocrine mediators of physical activity

### Exercise as an integrative endocrine challenge

Each exercise bout transiently disturbs homeostasis and activates coordinated endocrine responses that support substrate mobilization, cardiovascular adjustment, thermoregulation, water–electrolyte balance, tissue repair, among others. The acute endocrine response to exercise is highly dynamic, with hormonal changes varying according to the characteristics of the exercise bout and the individual. It is shaped by exercise mode, volume, intensity, training status, environmental stress, and energy availability (125, 126). Repeated exercise training does not abolish these acute endocrine responses; rather, it attenuates the endocrine perturbation elicited by a given absolute workload as physical fitness improves. Still, it usually makes the same external workload less stressful internally, so the hormonal cost of a given task declines as fitness improves (126).

Exercise recruits several classical endocrine glands in a coordinated and time-dependent manner. The adrenal medulla is among the most rapidly activated components, increasing EPI and NE release in close relation to exercise intensity and duration. These catecholamines support cardiovascular adjustment, hepatic glucose output, and adipose tissue lipolysis (127). The hypothalamic–pituitary–adrenal (HPA) axis is also engaged, particularly during prolonged or high-intensity exercise, leading to ACTH-dependent cortisol secretion from the adrenal cortex and contributing to substrate availability and stress adaptation (125). The anterior pituitary responds by increasing growth hormone (GH) secretion, a response that is strongly influenced by exercise intensity and metabolic stress (128). The posterior pituitary contributes through arginine vasopressin release, which helps preserve plasma volume and water balance during exercise, especially when thermal strain, dehydration, or prolonged effort are present (129). Pancreatic islets also participate in this endocrine adjustment, with a relative suppression of insulin secretion and stimulation of glucagon, thereby favoring glucose mobilization rather than storage during sustained energetic demand (130). Finally, regular exercise also modulates the renin-angiotensin-aldosterone system (RAAS). A recent meta-analysis demonstrated that chronic exercise training reduces circulating angiotensin II and aldosterone concentrations, suggesting that modulation of RAAS activity contributes to the cardiovascular adaptations associated with regular physical exercise (131).

### Skeletal muscle, adipose tissue, and other organs as exercise-responsive endocrine systems

Skeletal muscle acts as a reciprocal endocrine hub, exchanging myokine, metabolic, and mechanical signals with the heart and brain, closing a feedback loop that links central command, cardiac adaptation, and peripheral muscular remodeling into a single coordinated network.

Among non-classic endocrine tissues engaged by exercise, skeletal muscle remains the best characterized. The literature supports the view that muscle releases hundreds of myokines with autocrine, paracrine, and endocrine actions, and these signals participate in crosstalk with adipose tissue, liver, bone, heart, and brain (132). Interleukin 6 (IL-6) is the canonical example, but several other myokines, including irisin, myonectin, interleukin 15 (IL-15), decorin, and Follistatin-like 1 (FSTL1), have also been implicated in exercise-induced adaptations (133).

Adipose tissue is also an important endocrine organ during exercise, although its secretory response is strongly influenced by adiposity, energy balance, training status, and inflammatory tone. White and brown adipose depots release a broad repertoire of adipokines, including leptin, adiponectin, resistin, apelin, tumor necrosis factor alpha (TNF-α), and IL-6, that regulate appetite, insulin sensitivity, inflammation, vascular function, and substrate partitioning (134).

The liver and heart also participate in exercise-driven endocrine communication. Hepatokines such as Fibroblast Growth Factor 21 (FGF21), Angiopoietin-like protein 4 (ANGPTL4), fetuin-A, and follistatin respond to nutritional status and physical activity, and the exercise literature increasingly treats them as transducers of broader cardiometabolic benefit (135). Furthermore, natriuretic peptides produced by the heart are *bona fide* endocrine mediators with renal, vascular, and adipose actions, and they sit at the intersection of hemodynamic control and exercise-related lipid mobilization (136, 137).

### Endocrine mediators of metabolic responses to acute and chronic exercise

Acute exercise elicits a coordinated endocrine response that integrates multiple hormonal signals to regulate substrate mobilization and energy supply. At the onset of exercise, the decline in insulin relieves the inhibition of hepatic glucose output and adipose tissue lipolysis. In parallel, the rise in glucagon primarily supports hepatic glucose production, whereas catecholamines stimulate both hepatic glucose production and adipose tissue lipolysis, thereby increasing the availability of glucose and fatty acids for working muscle. Contracting skeletal muscle increases glucose uptake predominantly through contraction-mediated, insulin-independent mechanisms (138). Increased venous return and atrial stretch stimulated by physical exercise increase cardiomyocyte Atrial natriuretic peptide (ANP) release, which promotes adipose tissue lipolysis via NPR-A–cGMP–PKG signaling, complementing catecholamine action and increasing fatty acid availability for working muscle (139).

As exercise is prolonged and intramuscular glycogen stores decline, contracting skeletal muscle releases greater amounts of IL-6. This muscle-derived IL-6 may contribute to the maintenance of hepatic glucose output and the stimulation of adipose tissue lipolysis, thereby helping preserve substrate availability during sustained energetic demand (140, 141). Other muscle-derived factors may further fine-tune substrate handling during exercise. Myonectin links skeletal muscle activity to systemic lipid metabolism by favoring fatty acid uptake and oxidation in metabolic tissues (142), whereas BDNF, apelin, and IL-15 may support oxidative metabolism and glucose utilization within skeletal muscle (143, 144). However, compared with insulin withdrawal, glucagon, catecholamines, ANP, and IL-6 are better interpreted as modulatory or adaptive signals rather than primary acute regulators of hepatic glucose output or adipose tissue lipolysis (145).

FGF21 may further complement this response as an exercise-responsive hepatokine with predominantly lipid-modulating actions. Following acute exercise, its circulating levels rise, primarily due to splanchnic secretion driven by an increased glucagon-to-insulin ratio and augmented fatty acid flux from adipose tissue to the liver. In this context, while glucagon and catecholamines remain the principal acute regulators of hepatic glucose output and adipose tissue lipolysis, FGF21 likely functions as a secondary endocrine signal during prolonged or energetically demanding exercise, helping shift systemic metabolism toward greater fatty acid utilization and reduced hepatic lipid accumulation, while also supporting glucose homeostasis through effects on insulin sensitivity and peripheral substrate handling (146, 147). The liver also contributes to the acute regulation of lipid partitioning by releasing angiopoietin-like protein 4 (ANGPTL4) in response to elevated circulating fatty acids. During acute exercise, circulating ANGPTL4 is derived predominantly from the liver, with additional contributions from skeletal muscle and adipose tissue, which inhibits lipoprotein lipase in non-working tissues, thereby redirecting triglyceride-derived fatty acids toward contracting skeletal muscle and supporting an oxidative fuel selection (148).

When repeated across successive training sessions, the acute endocrine responses described above gradually remodel metabolic gene expression, receptor sensitivity, and tissue function, thereby contributing to the broad metabolic benefits of chronic exercise (149). The improvement in insulin sensitivity that accompanies chronic exercise training reflects the convergent hormonal action of multiple signals reinforced over weeks of exercise (150). With repeated training sessions, contraction-induced AMP-activated protein kinase (AMPK) activation works alongside exercise-responsive endocrine signals, particularly muscle-derived IL-6, liver-derived FGF21, and adipokines such as adiponectin, to enhance skeletal muscle glucose handling. Over time, this integrated response increases GLUT4 protein abundance and improves insulin-stimulated IRS–PI3K–Akt–AS160 signaling, allowing the same circulating insulin concentration to elicit a greater rate of glucose disposal (151, 152). This adaptation is reinforced by the recurrent post-exercise surges in GH and IGF-1, which support myofibrillar and mitochondrial protein remodeling, and by the cumulative attenuation of cortisol responses to submaximal effort, which reduces glucocorticoid-driven interference with insulin signaling in skeletal muscle and adipose tissue (125, 128). Moreover, training-induced upregulation of Guanylyl Cyclase/Atrial Natriuretic Peptide Receptor-A (ANP/GCA) receptor expression in skeletal muscle reinforces mitochondrial oxidative phosphorylation and insulin action (153). At the same time, the repeated lipolytic stimulation of adipose tissue by ANP progressively sensitizes Natriuretic Peptide Receptor A (NPR-A) pathways, reducing ectopic lipid accumulation, a well-established driver of insulin resistance (154).

The literature supports multiple endocrine and hormone-like signals as promoters of white adipose tissue (WAT) browning. Catecholamines are the best-established stimulus, acting through β-adrenergic receptors, predominantly β2-AR in humans (155) and β3-AR in rodents (156). Natriuretic peptides display reasonable mechanistic support through p38 MAPK/UCP1 signaling, with evidence in primary human adipocytes and *in vivo* murine models (157), although most robust *in vivo* evidence still comes from rodent studies, and their clinical relevance in healthy humans remains to be established (158).

Repeated bouts of exercise may increase FGF21, promoting hepatic and peripheral lipid oxidation, enhancing adiponectin signaling, and reducing ectopic lipid accumulation in the liver (146, 147). FGF21 regulates WAT browning via PGC-1α under specific conditions, and Fgf21-knockout mice exhibit impaired cold-induced browning (159). However, a pivotal study demonstrated that FGF21's metabolic effects *in vivo*, including weight loss and improved glucose homeostasis, are largely independent of UCP1 and beige adipocyte formation, suggesting that browning may be a circumstantial rather than mechanistically central effect of this factor (160). Irisin was originally identified as a PGC-1α-dependent myokine that drives white-fat browning and UCP1 expression in mice (161). Cell-based evidence of UCP1 upregulation in human adipocytes via the ERK/p38 MAPK pathway exists (162), but its physiological relevance in humans *in vivo* remains unresolved.

Exercise training commonly lowers leptin and can increase adiponectin, especially in populations with diabetes, prediabetes, overweight, or larger fat-mass losses, but the effect is not universal, and older studies found little change when adiposity remained stable (163, 164). Adiponectin is generally insulin-sensitizing, anti-inflammatory, and vasoprotective, whereas leptin integrates energy stores with appetite, sympathetic activity, and inflammation (165). Together, these effects help improve metabolic flexibility, insulin sensitivity, and lipid handling, thereby contributing to the broader cardiometabolic benefits of chronic exercise training.

Successive exercise sessions elicit transient hormonal and myokine responses that cumulatively activate transcriptional programs involved in mitochondrial biogenesis and oxidative metabolism, with Peroxisome proliferator-activated receptor gamma coactivator 1-alpha (PGC-1α) acting as a central regulatory hub (166). In this context, recurrent activation of the GH/IGF-1 axis (167), cardiac natriuretic peptides (139, 154), adiponectin (165), and FGF21 (168) may act together with muscle-derived signals such as musclin/osteocrin (169), neurturin (170) to reinforce PGC-1α-dependent oxidative gene programs. Over time, this coordinated endocrine and myokine-driven remodeling supports the expansion of mitochondrial content and improves oxidative capacity in skeletal muscle, thereby contributing to greater metabolic flexibility, enhanced lipid oxidation, and improved whole-body energy homeostasis with chronic exercise training.

Mitochondrial content and VO_2_max are also stimulated by exercise training, and the extent of effects is determined by the initial fitness status of the individuals (171). Therefore, mitochondria are powerhouse organelles that provide energy in the form of ATP currency, controlling the levels of ROS and calcium signaling, which are essential for maintaining body activity during exercise. Thus, exercise and mitochondria are in a close relationship and influence each other (172).

The increased energy demand during physical activity may also lead to reductions in intracellular pH and increased production of reactive oxygen species in skeletal muscle (173). Intense exercise induces a transient decrease in blood and skeletal-muscle pH, reflecting increased H⁺ production from anaerobic glycolysis and lactate accumulation, with recovery occurring over minutes to hours post-exercise depending on intensity and buffering. Maintaining intracellular pH homeostasis and redox status in muscle is critical to preserve tissue homeostasis (e.g., contractile capacity, tissue integrity, cell signaling, and others) (174). Thus, compounds affecting these parameters can impact mitochondrial health (175). Intramuscular ATP stores sustain maximal contraction for approximately 1–2 s before phosphocreatine becomes the predominant immediate energy source. Several ATP resynthesis pathways are engaged during vigorous exercise, both anaerobic, substrate-level phosphorylation, and aerobic metabolism, which is highly dependent on the oxygen delivered by the integrated cardiovascular respiratory systems to the skeletal muscle (172).

Mitochondrial dynamics relies on integrated mechanisms that contribute to maintain cellular integrity and meet changing metabolic demands, by adjusting the shape, size, number, and location of mitochondria (176). Mitochondrial fusion merges individual mitochondria to expand the network to enhance respiratory efficiency, whereas mitochondrial fission splits them into smaller units, allowing for isolation of damaged components or organelle multiplication (106). *De novo* mitochondria biogenesis depends on the production of new proteins and DNA, a process mainly controlled by the master regulator PGC-1α (177). Dysfunctional mitochondria are selectively removed through a quality-control mechanism called mitophagy, maintaining a healthy organelle pool (178). These mechanisms cooperate to ensure a high-functioning pool of organelles, optimized for ATP supply.

Thus, metabolic benefits of chronic exercise training, including improved insulin sensitivity, enhanced oxidative capacity, more favorable body composition, and lower cardiometabolic risk, can be understood as the cumulative outcome of repeated, transient endocrine responses that, although short-lived and context-dependent at each exercise bout, progressively shape systemic metabolic adaptation.

## Nutritional strategies, dietary supplementation, and exercise performance

Throughout this section, exercise training is treated as the primary intervention and nutrition as adjuvant. Priority is therefore given to nutrients with a plausible mechanistic point of convergence on exercise-evoked adaptation, namely creatine monohydrate and long-chain omega-3 fatty acids, whereas whole dietary patterns are summarized only to the extent that they modify the response to training.

The interface between nutritional intake and exercise adaptation extends well beyond caloric balance. Specific nutrients and dietary patterns modulate signaling cascades that govern muscle protein synthesis, mitochondrial biogenesis, neuroplasticity, and systemic inflammation, all of which determine whether a given training stimulus translates into measurable gains in performance and well-being. This section examines ergogenic [Greek ergon (work) + gennan (to produce)] supplements supported by robust evidence and discusses how distinct dietary frameworks interact with exercise physiology.

Creatine monohydrate remains the most extensively validated ergogenic supplement in sports nutrition, which is any substance taken to enhance physical performance, strength, endurance, or recovery beyond what would be achieved through training alone. Its mechanism is direct: exogenous creatine loading saturates the intracellular phosphocreatine pool, accelerating ATP regeneration through the creatine kinase shuttle during high-intensity, short-duration contractions (179). At maintenance doses of 3–5 g·day^−1^, creatine supplementation reliably augments maximal strength, power output, and the capacity to sustain repeated high-intensity efforts across diverse populations (180). The functional consequence for hypertrophy is indirect but consistent: by permitting higher training volumes and greater mechanical tension per session, creatine amplifies the cumulative stimulus for myofibrillar protein accretion. Meta-analytic data from trials employing direct imaging endpoints (ultrasound or MRI) confirm small but significant gains in muscle thickness and cross-sectional area when creatine is combined with progressive RT lasting eight weeks or longer (181).

The clinical relevance of creatine has expanded substantially in recent years, most notably into the domain of brain health. The brain, despite comprising roughly 2% of body mass, consumes approximately 20% of resting metabolic energy, and phosphocreatine buffering is integral to maintaining neuronal ATP homeostasis during periods of heightened demand (182). A meta-analysis of 16 randomized controlled trials encompassing 492 adults aged 20–76 years found that creatine supplementation significantly improved memory performance (SMD = 0.31, 95% CI: 0.18–0.44), with effects most pronounced in females, individuals aged 18–60, and those with pre-existing cognitive conditions (183). High-dose creatine supplementation (10 g·day^−1^) may be required in some individuals to meaningfully increase brain creatine content, given the limited permeability of the BBB to creatine (183). A 6-week trial of 10 g·day^−1^ creatine monohydrate in healthy adults demonstrated improvements in word recognition and picture recognition tasks, though not all cognitive domains responded uniformly.

The mechanistic basis for this synergy lies in the spatial, rather than merely temporal, function of the creatine kinase circuit. Creatine enters the brain through the sodium-dependent transporter SLC6A8 and is also synthesized locally by AGAT and GAMT, and the reversible transfer of a phosphoryl group between ATP and creatine allows high-energy phosphates to move from mitochondria to sites of rapid ATP consumption in the cytosol, sustaining local ATP/ADP ratios where diffusion of adenine nucleotides alone would be too slow. This matters for exercise-evoked plasticity because the processes that exercise recruits, namely repetitive high-frequency firing, glutamate cycling, dendritic remodeling and the protein synthesis that consolidates long-term potentiation, are among the most ATP-expensive events in neural tissue. Consistent with an energy-buffering role that becomes visible only under load, the cognitive effects of creatine supplementation are most apparent under acute metabolic stress such as sleep deprivation or hypoxia and in populations with reduced baseline brain creatine, rather than in unstressed young adults (184, 185). This same literature identifies the principal limitation of the strategy: standard muscle-oriented doses raise muscle creatine reliably but brain creatine only modestly, and the protocol required to increase brain creatine content has not been established.

Omega-3 fatty acids, eicosapentaenoic acid (EPA) and docosahexaenoic acid (DHA) serve as precursors for endocannabinoid synthesis and are produced by essential polyunsaturated fatty acids (PUFAs), making them crucial for optimizing exercise-induced endocannabinoid responses (186). These fatty acids are incorporated into cell membranes, where they can be converted into endocannabinoids on demand (Figure 5). A diet rich in omega-3s from sources like fatty fish, flaxseeds, chia seeds, and walnuts may enhance the endocannabinoid response to exercise, potentially amplifying the mood-boosting and analgesic effects (188). Evidence suggests that combining omega-3 supplementation with regular exercise may produce greater improvements in mood and cognitive function than either intervention alone.

**Figure 5:**
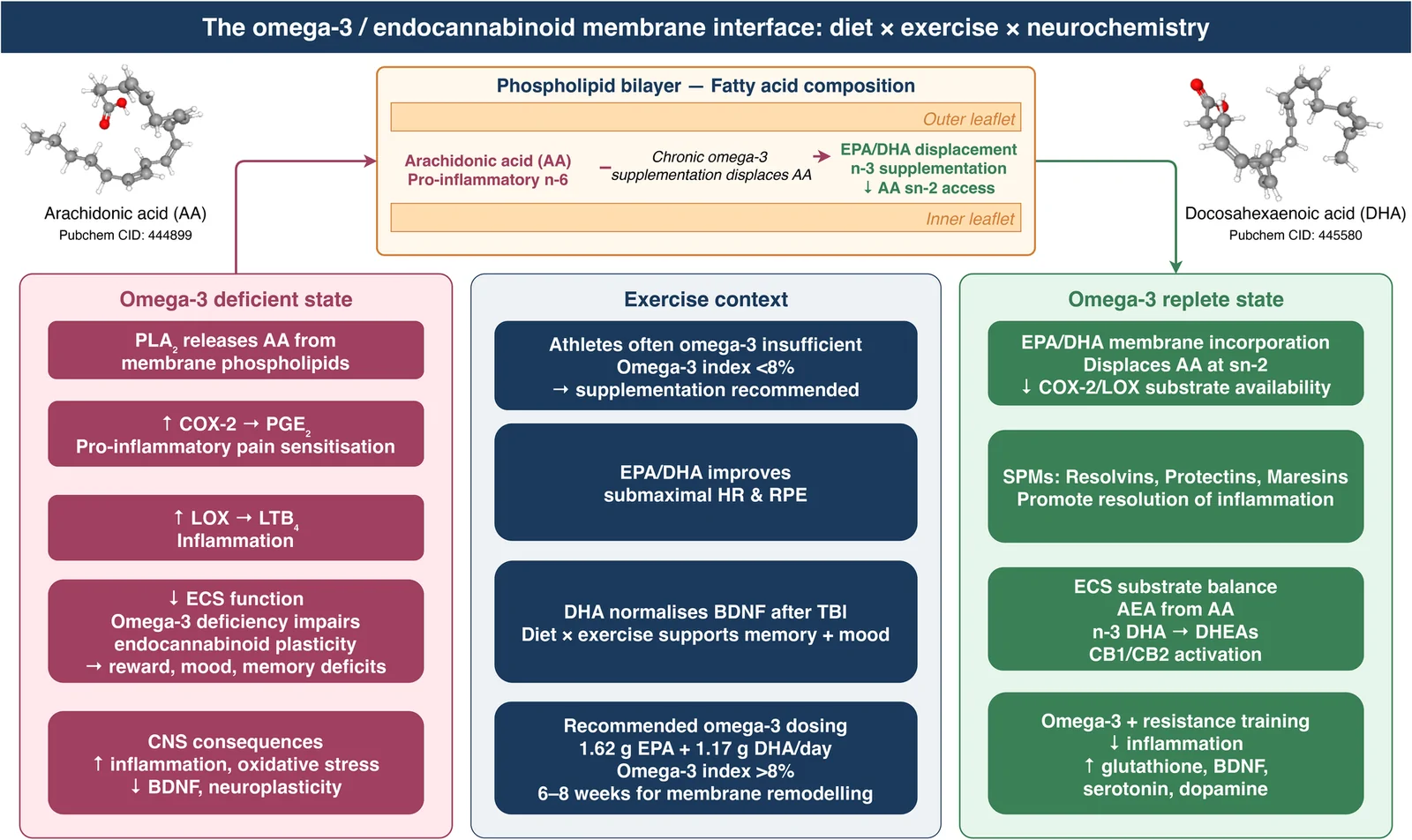
Omega-3 fatty acids, EPA and DHA serve as precursors for endocannabinoid synthesis and are long-chain n-3 PUFAs derived from the essential precursor α-linolenic acid or obtained directly from marine sources, making them potentially important for optimizing exercise-induced endocannabinoid responses. These fatty acids are incorporated into cell membranes, where they can be converted into endocannabinoids on demand. While omega-3 supplementation reliably attenuates indicators of exercise-induced muscle damage and delayed-onset muscle soreness, the evidence for direct enhancement of maximal aerobic capacity or strength output is doubtful (187). Omega-3 supplementation is therefore best conceptualized as a recovery-facilitating strategy rather than a primary ergogenic aid. The conversion of EPA and DHA into N-acylethanolamines and the sn-2 displacement of arachidonic acid in membrane phospholipids are established biochemical pathways. The performance-related items in the central panel are of much weaker standing: reductions in submaximal heart rate and rating of perceived exertion have been reported, but omega-3 supplementation does not reliably increase maximal aerobic capacity or maximal strength (187), and the dosing shown reflects the regimens most often tested rather than an established optimum. Attenuation of muscle-damage markers and delayed-onset soreness is the most consistently reproduced effect and is the basis for presenting supplementation as a recovery strategy.

EPA and DHA are incorporated into phospholipid bilayers upon chronic supplementation, displacing arachidonic acid and shifting the eicosanoid profile toward less inflammatory mediators (189). Membrane remodeling reduces the capacity for cyclooxygenase-2 (COX-2) and 5-lipoxygenase to generate prostaglandin E_2_ and leukotriene B_4_, while simultaneously providing substrate for the biosynthesis of specialized pro-resolving mediators, including resolvins, protectins, and maresins, that actively drive the resolution phase of inflammation rather than merely suppressing its initiation (186, 190).

Two mechanisms link omega-3 status directly to the plasticity that exercise evokes. The first is convergence on BDNF signaling. In rats, twelve days of DHA supplementation combined with voluntary wheel running raised hippocampal pro-BDNF and mature BDNF, phosphorylated CREB, synapsin I, Akt and CaMKII, and improved spatial learning, in every case to a greater extent than either intervention alone (191); the same combination acts on the synaptic membrane itself, elevating syntaxin-3, GAP-43 and the NMDA receptor subunit NR2B (192). Since Akt and CaMKII are the effectors through which BDNF acts on synaptic plasticity, DHA availability and exercise converge on a shared node rather than acting in parallel. The second is the generation of a bioactive DHA derivative: N-docosahexaenoylethanolamine (synaptamide) activates the adhesion receptor GPR110 (ADGRF1), triggering cAMP-dependent neurite outgrowth and synaptogenesis (193). This pathway is best characterized in developing neurons, in which GPR110 expression is high and declines after birth, and its contribution to plasticity in the adult exercising brain remains hypothetical. Both mechanisms rest on rodent and *in vitro* evidence and provide a rationale for the adjuvant role of omega-3 rather than proof of an effect in exercising humans.

The International Society of Sports Nutrition concluded that athletes are frequently omega-3 insufficient, with omega-3 index values well below the cardioprotective threshold of 8% (194). A 2025 randomized trial in 30 physically active males demonstrated that combined omega-3 supplementation (1,620 mg EPA + 1,170 mg DHA daily) during eight weeks, together with RT, significantly reduced circulating IL-6, tumor necrosis factor (TNF-α) and malondialdehyde (a lipid peroxidation marker), while glutathione levels were increased relative to the training-only control (195). The same study reported elevations in BDNF, 5-HT and DA in the supplemented group, pointing to CNS effects that extend beyond peripheral anti-inflammatory action.

The relationship between omega-3 fatty acids and the endocannabinoid system deserves particular attention. EPA and DHA serve as biosynthetic precursors for endocannabinoid-related N-acyl ethanolamine's and dietary omega-3 status profoundly influences endocannabinoid tone: lifelong omega-3 deficiency in mice abolishes endocannabinoid-mediated long-term synaptic depression in the prefrontal cortex and nucleus accumbens by uncoupling CB₁ receptors from their effector Gᵢ/ₒ proteins (196). Indeed, omega-3 deficient-fed mothers had decreased CB₁ receptor protein levels in the cerebral cortex and increased CB_2_ receptor levels in both the cerebral cortex and hippocampus. Furthermore, postnatal day 2 neonates from mothers with maternal omega-3 deficiency showed reduced CB₁ receptor levels in the hippocampus while showing increased CB_2_ receptor levels (197). Maternal omega-3 supplementation increased synaptic markers and PKA phosphorylation in the cortex and ERK phosphorylation in the hippocampus of postnatal rats. These findings provide a plausible mechanistic link between dietary fatty acid composition and the endocannabinoid-mediated analgesic, anxiolytic, and mood-related responses associated with exercise.

The individual amino acids commonly related to exercising populations occupy different layers of evidential support. Leucine stands as the main activator of the mechanistic target of rapamycin complex 1 (mTORC1) signaling pathway, which governs translational initiation of muscle protein synthesis (MPS). Maximizing the acute muscle protein synthesis response in healthy adults requires reaching a per-meal leucine threshold of roughly 2–3 g (198), a target generally met by ingesting approximately 20 g of high-quality protein in young adults, with higher doses (up to ∼40 g) required in older adults and when whole-body protein turnover is considered (199, 200). Beyond a daily intake of approximately 1.6 g·kg^−1^·day^−1^ (95% CI 1.03–2.20), however, additional protein confers progressively smaller gains in lean mass during RT (201). The practical ceiling identified by these data has shaped current protein recommendations and, by extension, the rationale for supplementing with branched-chain amino acids (BCAAs) or isolated essential amino acids (EAAs) only when whole-food protein intake is suboptimal.

Glutamine, the most abundant free amino acid in skeletal muscle and plasma, has been proposed as an immunomodulatory supplement for athletes undergoing heavy training. The hypothesis rests on the observation that prolonged exhaustive exercise transiently depresses plasma glutamine, potentially compromising lymphocyte and macrophage function during the so-called “open window” of post-exercise immunosuppression (202). Supplementation trials, however, have produced mixed results. While glutamine provision (typically 5–10 g post-exercise) may attenuate the incidence of upper respiratory tract infections in endurance athletes (203), its effects on direct immune cell function markers are modest and context-dependent. Its role may be more consequential for gut epithelial integrity, where glutamine serves as a preferred fuel for enterocytes, and where exercise-induced intestinal permeability (“leaky gut”) can trigger systemic endotoxemia during prolonged effort (204).

Lysine has received less attention in the sport nutrition literature but holds relevance through its obligate role in collagen crosslinking and carnitine biosynthesis. Adequate lysine availability is required for hydroxylysine formation and subsequent collagen maturation via lysyl oxidase-mediated crosslinking, processes critical for tendon and connective tissue remodeling during RT (205). Lysine, together with methionine, is also a precursor for endogenous carnitine synthesis, which facilitates long-chain fatty acid transport into the mitochondrial matrix for β-oxidation, a process of relevance during prolonged moderate-intensity exercise. Populations consuming predominantly cereal-based diets, where lysine is the first limiting amino acid, may be at particular risk for suboptimal collagen turnover and impaired fatty acid metabolism.

### Diet, neuroprotection and cardiovascular co-benefits

The Mediterranean diet (MeDi) is characterized by high consumption of fruits, vegetables, legumes, whole grains, nuts, olive oil, and fish, moderate wine consumption, and reduced intake of red meat and processed foods. This dietary pattern is consistently associated with improvements in cardiovascular health, including reductions in LDL cholesterol, systemic inflammation, and endothelial dysfunction, as demonstrated in large-scale trials such as the PREDIMED trial (206). These adaptations are directly relevant to exercise physiology, as improved endothelial function enhances peripheral oxygen delivery, while reduced inflammation may accelerate post-exercise recovery.

Adherence to MeDi is also associated with reduced risk of cognitive decline and Alzheimer's disease (207). These beneficial effects are believed to involve multiple mechanisms, including inhibition of amyloid-β aggregation and neuroinflammation by olive oil polyphenols (e.g., oleocanthal and hydroxytyrosol), maintenance of synaptic membrane integrity through omega-3 fatty acids, and modulation of the gut-brain axis via short-chain fatty acids derived from dietary fiber, influencing vagal signaling and systemic cytokine regulation (208).

The MIND diet, combining Mediterranean and Approaches to Stop Hypertension (DASH) principles, emphasizes specific neuroprotective food groups and has been associated with improvements in cognitive performance (e.g., working memory, processing speed, and verbal learning) alongside reductions in plasma homocysteine (209). When combined with regular physical activity, these dietary patterns likely exert additive effects through convergent mechanisms involving increased BDNF expression and suppression of NF-κB-mediated neuroinflammation.

Carbohydrate restriction below 50 g·day^−1^ shifts substrate use toward fatty acid oxidation and hepatic ketogenesis, raising circulating β-hydroxybutyrate, which acts both as a fuel and as a signaling molecule that inhibits histone deacetylases, activates HCA2 receptors and suppresses the NLRP3 inflammasome (210). Ketosis has been associated with improved cognitive performance in Alzheimer's disease, although the effect appears to reverse on diet cessation (211). For exercise, the trade-off is intensity-dependent: ketogenic adaptation increases fat oxidation during submaximal work (212) but raises the oxygen cost of ATP production relative to glucose, impairing efforts that depend on high glycolytic flux (213). The ketogenic diet may therefore suit metabolically compromised individuals training at moderate intensity and disadvantage athletes performing high-intensity or intermittent work.

Intermittent fasting protocols induce a comparable glucose-to-ketone metabolic switch after 12–36 h, engaging AMPK, sirtuin and autophagy pathways (214). Their interaction with training is protocol-dependent: a 2025 meta-analysis of 34 randomized trials found that time-restricted eating increased maximal oxygen uptake (SMD = 1.32, *p* = 0.001) whereas Ramadan fasting, which also restricts fluid intake during daylight, reduced it (SMD = −2.20, *p* < 0.001), with no significant effect of either protocol on muscle strength or anaerobic capacity (215). Resistance training performed fasted or fed yields equivalent gains in fat-free mass, hypertrophy and maximal strength, indicating that total daily energy and macronutrient intake, rather than pre-exercise feeding status, drives chronic adaptation (216, 217).

## Anabolic-androgenic steroids in recreational exercise contexts

The non-medical use of anabolic-androgenic steroids (AAS) has expanded well beyond competitive sport into the broader fitness community. A global meta-analysis estimated the lifetime prevalence of AAS use at 3.3% among the general population, with rates exceeding 10% among male gym-goers (218). Motivations extend beyond performance to encompass body image ideals, with muscularity-oriented concerns driving initiation in a substantial proportion of users (219). Notably, the trajectory from legal dietary supplements (protein powders, creatine, pre-workout formulas) to AAS has been described as a “gateway” phenomenon, in which progressive dissatisfaction with natural training outcomes and normalization of ergogenic aid use within gym culture lower the perceived threshold for illicit steroid initiation. The cardiovascular consequences of chronic supraphysiological AAS exposure are increasingly recognized as a major source of morbidity, with some structural and functional alterations potentially persisting even after AAS discontinuation. A 2025 meta-analysis quantified AAS-associated increases in systolic and diastolic blood pressure of 12.4 and 8.1 mmHg, respectively, alongside significant reductions in HDL cholesterol and elevations in LDL cholesterol (220). These hemodynamic and lipid perturbations accelerate atherogenesis and predispose to premature coronary artery disease. Beyond conventional risk factors, AAS exert direct myocardial toxicity: chronic use promotes left ventricular hypertrophy, impairs diastolic function, and induces interstitial fibrosis through androgen receptor overactivation, oxidative stress, and dysregulation of the renin-angiotensin-aldosterone system (221). A large Danish registry study of AAS users found significantly elevated rates of cardiovascular disease relative to non-users, with hazard ratios remaining significant after adjustment for conventional confounders (222).

Endocrine disruption is an inevitable consequence of exogenous androgen administration. Hypothalamic-pituitary-gonadal axis suppression manifests as hypogonadotropic hypogonadism, with profound reductions in endogenous testosterone, FSH, and LH that may persist for months to years after cessation of use, particularly following prolonged or high-dose regimens. Spermatogenesis is severely impaired, and clinical infertility is a well-documented sequela (223). In women, virilizing effects, including voice deepening, hirsutism, and clitoral hypertrophy, are often partially irreversible. Hepatic consequences range from cholestatic jaundice to peliosis hepatis and, in rare cases, hepatocellular carcinoma, particularly with 17α-alkylated oral compounds.

The psychiatric dimensions of AAS use are clinically significant and bidirectional. Supraphysiological androgen levels can precipitate irritability, aggression (“roid rage”), mania, and psychotic features during cycles of use, while withdrawal phases are frequently accompanied by depression, anhedonia, and suicidal ideation (224). A body of neuroimaging evidence suggests that long-term AAS exposure is associated with reduced cortical thickness and alterations in white matter integrity, though causality remains difficult to establish in observational designs (225).

In rodent models, regular exercise training upregulates endogenous antioxidant enzymes and enhances mitochondrial quality control through PGC-1α-dependent pathways, supraphysiological exposure to exogenous androgens, such as the nandrolone decanoate ester, produces the opposite profile, characterized by increased reactive oxygen species generation, depletion of superoxide dismutase and glutathione peroxidase, and mitochondrial dysfunction in cardiac, hepatic, and renal tissue (226). In rat cardiac myocytes, this AAS-induced oxidative stress engages the TLR4/NF-κB/NLRP3 inflammasome axis and mitochondrial apoptotic signaling, promoting the fibrotic remodeling that underpins AAS-associated cardiomyopathy (227).

## Exercise and immunity

For more than a century, it has been recognized that physical activity can modulate immune responses. The first clue described that exercise could modulate immunity was a leukocytosis in subjects who run a marathon, which returned to basal levels after a resting period (228). Later, between 30's and 50's, the work developed by Dr Hans Selye, known as the Father of Stress Research, stated a new syndrome produced by diverse nocuous agents (later called General Adaptation Syndrome), including exhausting exercise, in which adrenal hypertrophy, thymic atrophy, and gastric ulcers where observed (229).

It is well established that exercise modulates immune cells numbers, depending on the exercise intensity, duration and on the time analyzed. Basically, there is an increase in the numbers of leukocytes in the blood during exercise, followed by a marked reduction in lymphocytes within a few hours. It has been proposed that catecholamines produced immediately after beginning exercise and cortisol, later (in hours), are responsible for the cellular alterations, also referred to as bi-phasic model (230). The effect of vigorous exercise on leukocytes subpopulations is documented. It was demonstrated an increase in neutrophils, total lymphocytes (CD4^+^, CD8^+^, B cells, and NK cells), and in apoptotic lymphocytes during exercise, followed by a reduction in counts in all lymphocytes subpopulations and an increase in neutrophils and apoptotic lymphocytes after the activity (231).

There is overwhelming evidence supporting the health benefits of physical activity. Regular physical activity has been associated with a marked reduction in the risk for premature mortality and reduces the risks for more than 25 chronic medical conditions (232). Also, it has been reported that ∼2 h of moderate exercise per day is associated with a 29% reduction in risk of developing up upper respiratory tract infection compared with sedentary individuals (233). Furthermore, there is convincing epidemiological evidence to support the health benefits of exercise. A systematic review published in 2017 evaluated 16 systematic reviews and/or meta-analyses within almost 2 million participants, and in all studies it was demonstrated a dose–response relationship between physical activity and health outcomes (234).

An important point of discussion concerning the effect of exercises on immunity refers to the activity intensity and duration. Indeed, the work developed by Dr Nieman and colleagues established fundamental principles about how exercise modulates immunity. Based on the work of professor's Nieman group, it is postulated that moderate exercise reduces the incidence of respiratory infections and strengthens immune surveillance, while intense and prolonged exertion can temporarily suppress the body's defenses (235). Supporting the idea that prolonged exercise can be deleterious to the immune system, it was observed that symptoms of upper respiratory tract infection occurred in 33% of ultramarathon runners (compared to 15% of controls (236). Corroborating, it was also observed that 47% of marathon runners reported upper respiratory tract infection symptoms days after the run, indicating that lung environment is more susceptible to infections after an intense physical activity (237). Furthermore, it has been confirmed that elite athletes have a higher rate of upper respiratory illness, compared to recreationally competitive athletes or with sedentary controls (238). Several possibilities were raised to answer the mechanisms related to the exercise-induced impaired immune function, such as an increase in stress hormones, the release of immature leukocytes from the bone marrow to the circulation, a decrease in glutamine serum levels, and an increase in ROS production. The basis of the increased incidence of infection in athletes is probable due to multiples reasons, such as physical, psychological, environmental, and nutritional stressors (202). The susceptibility observed after vigorous exercise is partly explained by observations of salivary IgA levels, inasmuch lower concentrations of salivary IgA have been reported in cross-country skiers after a race, after long-duration ergometer cycling, intense swimming, running, and after progressive treadmill running to exhaustion (231).

On the other hand, the concept that extenuating exercise is immunosuppressive has been debated. Regular physical activity and/or frequent structured exercise reduces the incidence of many chronic diseases in older age, including viral and bacterial infections, as well as non-communicable diseases such as cancer and chronic inflammatory disorders. However, observed immunosuppression after prolonged exercise might be an artefact, mainly because none of the self-reported infections were clinically confirmed by laboratory analyses (e.g., molecular or microbiological techniques, such as PCR or bacterial cultures (239). Indeed, it was observed that among 37 episodes of upper respiratory tract infection reported by athletes, only 11 of these (30%) had a positive laboratory diagnosis (238).

Despite the discussion regarding whether there is or not a period of immunosuppression after an exhausting physical activity, there is a universal agreement that regular exercises are beneficial to the immune system as well it mitigates ageing process.

### Effects of exercise on immune function

One of the best-characterized immunological responses to exercise is the transient modulation of cytokine production. The first evidence that exercise induced a cytokine release was reported by Cannon and Kluger (240), in a study in which they injected the plasma obtained from human subjects after exercise into the peritoneal cavity of rats and observed the elevation of rectal temperature, suggesting the presence of pyrogens in the plasma, later characterized as IL-1, IL-6, and TNF-α.

Amongst cytokines members, an increase in plasma levels of TNF-α, MIP-1β (CCL4), IL-1β; IL-6, IL-8, IL-10, and IL-1-receptor antagonist (IL-1ra) is described both during and after exercise (231). Concerning the origin of IL-6 production observed during exercise, it is accepted that major part of this cytokine found in plasma has the contracting muscle fibers as source (241). It was observed that individuals who started treadmill running, the serum IL-6 level was significantly enhanced 30 min after, with the peak at the end of 2.5 h of running time (242). In addition to IL-6, skeletal muscle is also recognized as source of IL-8 and IL-15 (243).

Regarding the exercise effects on resistance to infections, an increased number of upper respiratory tract infection symptoms has been reported in the days after strenuous exercise, while moderate training reduces the number of symptoms, reviewed by Pedersen and Hoffman-Goetz (231). On the other hand, virus-induced myocarditis was aggravated by exercise, given that it was demonstrated that swimming during the initial phase of Coxsackie B_3_ virus infection increased mortality from 5.5% to 50% (244).

Curiously, the role for Lipopolysaccharide (LPS) endotoxin as a player during and after exercise has been raised by few studies, based on its well know capacity to cross gut mucosa and reach the circulation, triggering inflammation. It was demonstrated that the majority (>80%) of athletes who participate in the ultra-run (81 km) event had plasma endotoxin concentrations above 0.1 ng/mL, considered the acceptable upper limit (245). Similar observation was found in athletes who took part in a triathlon competition (246). However, no increase in endotoxin levels were observed in cyclists after a 100-miles ride that it was not the cause of post exertion illness and unrelated to rhabdomyolysis (247). It is not understood the reasons for the divergence found between runners and cyclists, which implies that further studies are needed to assert the participation of LPS translocation during exercise.

#### Wearable biosensor frameworks and multi-omics integration: toward multi-scale quantification of exercise-induced adaptation

Exercise physiology is converging on two complementary technological frontiers that together promise multi-scale quantification of exercise-induced adaptation. Wearable biosensors have moved well beyond simple activity tracking toward integrated frameworks that combine physiological, kinematic, biochemical, and dynamic signals with training-relevant metrics (248), yet consumer-grade devices remain limited by measurement interference, inconsistent validation standards, and an inability to fully capture inter-individual variability—limitations that future advances in optical and spectroscopic sensing may help resolve (249), including emerging biologically derived materials such as microbial protein nanowires. In parallel, multi-omics approaches—conceptualized through frameworks such as “enduromics” and “resistomics”—are extending analysis from the systemic to the molecular level, integrating genomic, epigenomic, transcriptomic, proteomic, and metabolomic data across blood, urine, and extracellular vesicles to reveal the distinct molecular signatures of endurance vs. RT, with growing application in personalized exercise prescription for both clinical and non-clinical populations (250). This molecular resolution is already proving valuable in cardiac rehabilitation, where biomarkers such as interleukin-6, C-reactive protein, and lactate kinetics track patient-specific recovery trajectories, and where real-time wearable metabolite tracking could enable adaptive, feedback-driven rehabilitation protocols (251). The central message is that wearable biosensing and multi-omics profiling are not competing but complementary technologies: wearables supply continuous, ecologically valid physiological surveillance, while omics tools supply the mechanistic depth needed to interpret that data biologically. Realizing their combined potential for individualized rehabilitation, however, depends on overcoming persistent translational barriers—standardizing validation protocols, reducing the cost and complexity of multi-omics profiling, and developing simplified workflows compatible with the short timeframes typical of clinical rehabilitation programs (249, 251). Taken together, wearable biosensing and multi-omics profiling represent complementary rather than competing technologies.

#### Safety and individualization of exercise

Regarding safety considerations and exercise modalities differences, despite the broad physiological and psychosocial benefits reviewed, the translation of exercise-induced adaptations into meaningful HRQoL gains depends critically on appropriate individualization and safety screening. Physical activity must account for age, baseline cardiorespiratory and musculoskeletal fitness, comorbidities (particularly cardiovascular, metabolic, and orthopedic conditions), medication use, and prior training history, since the same absolute workload can represent a low physiological stimulus in a trained individual and an excessive cardiovascular or orthopedic load in a sedentary or clinically compromised one. HIIT, while efficient for improving VO_2_max and glycemic control, needs caution and, where indicated, medical clearance and supervised progression in populations with uncontrolled hypertension, coronary artery disease, or advanced frailty, given the acute hemodynamic and myocardial oxygen demand associated with vigorous effort. Similarly, RT programming should respect the principles of progressive overload and specificity within individually tolerated ranges to minimize injury risk while preserving the mechanical tension necessary for adaptation. Rather than applying uniform protocols, exercise interventions should therefore be customized—in modality, intensity, volume, and progression rate—to the individual's functional capacity and health status, with regular reassessment, since this individualized, safety-conscious approach is what ultimately allows the neurobiological, endocrine, cardiovascular, and muscular adaptations discussed throughout this review to translate into sustainable, real-world improvements in QoL.

## Conclusion

Evidence collected here is variable in strength or in the degree of causal inference it supports. Randomized controlled trials and meta-analyses deliver comparatively robust support for the effects of aerobic exercise on cardiovascular fitness, cognition, and mood, particularly when performed at intensities at or above 65% of VO_2_max, two to three sessions per week, for at least 40 min (64). On the other hand, RT's neuroprotective action acts primarily on training frequency (at least three sessions per week) in humans rather than on intensity, volume, or program duration, and operates mainly through IGF-1 rather than BDNF; its effects on neuropathological markers such as β-amyloid and neurofibrillary tangles remain small and inconsistent across studies, probably due to heterogeneity in population, protocol, and biomarker assay than to a true absence of biological effect (69). HIIT and combined training protocols occupy an intermediate position: despite documented efficiency for VO_2_max and glycemic control, their dose–response relationships remain incompletely characterized, and most supporting evidence derives from short trials (12 weeks to 6 months) in relatively healthy or moderately impaired populations, which limits confidence in extrapolating these findings to long-term, real-world, or clinically complex settings.

Several mechanistic connections discussed here were established in animal models rather than in humans: the myokine-mediated pathway linking peripheral muscle contraction, via irisin and cathepsin B, to central BDNF induction rests primarily on rodent and *in vitro* evidence. Whether these molecules cross the human BBB and contribute to cognitive gains remains still to be confirmed. Comparable uncertainty restricts the field's ability to define a single “optimal dose” of exercise: prescriptions effective for cardiovascular or metabolic outcomes do not necessarily coincide with those optimal for neurotrophic or cognitive benefit, and the continued absence of harmonized HRQoL instruments, standardized biomarker panels, and long-duration trials constrains direct comparison across modalities. Resolving these gaps, together with the safety and individualization considerations discussed above for higher-intensity and resistance modalities in clinically vulnerable populations, will require dose-ranging trials with standardized outcomes, longer follow-up, and mechanistic work bridging preclinical and human models, before exercise prescription for QoL can move from broad, evidence-informed recommendation toward individualized, evidence-based dosing.
